# Antiseizure and Neuroprotective Efficacy of Midazolam in Comparison with Tezampanel (LY293558) against Soman-Induced Status Epilepticus

**DOI:** 10.3390/toxics10080409

**Published:** 2022-07-22

**Authors:** Taiza H. Figueiredo, Vassiliki Aroniadou-Anderjaska, Volodymyr I. Pidoplichko, James P. Apland, Maria F. M. Braga

**Affiliations:** 1 Department of Anatomy, Physiology, and Genetics, F. Edward Hébert School of Medicine, Uniformed Services University of the Health Sciences, Bethesda, MD 20814, USA; taiza.figueiredo.ctr@usuhs.edu (T.H.F.); vanderjaska@usuhs.edu (V.A.-A.); volodymyr.pidoplichko.ctr@usuhs.edu (V.I.P.); 2 Department of Psychiatry, F. Edward Hébert School of Medicine, Uniformed Services University of the Health Sciences, Bethesda, MD 20814, USA; 3 Neuroscience Branch, U.S. Army Medical Research Institute of Chemical Defense, Aberdeen Proving Ground, Aberdeen, MD 21010, USA; James.p.apland.civ@mail.mil

**Keywords:** soman, seizures, neuroprotection, amygdala, midazolam, tezampanel

## Abstract

Acute exposure to nerve agents induces status epilepticus (SE), which can cause death or long-term brain damage. Diazepam is approved by the FDA for the treatment of nerve agent-induced SE, and midazolam (MDZ) is currently under consideration to replace diazepam. However, animal studies have raised questions about the neuroprotective efficacy of benzodiazepines. Here, we compared the antiseizure and neuroprotective efficacy of MDZ (5 mg/kg) with that of tezampanel (LY293558; 10 mg/kg), an AMPA/GluK1 receptor antagonist, administered 1 h after injection of the nerve agent, soman (1.2 × LD_50_), in adult male rats. Both of the anticonvulsants promptly stopped SE, with MDZ having a more rapid effect. However, SE reoccurred to a greater extent in the MDZ-treated group, resulting in a significantly longer total duration of SE within 24 h post-exposure compared with the LY293558-treated group. The neuroprotective efficacy of the two drugs was studied in the basolateral amygdala, 30 days post-exposure. Significant neuronal and inter-neuronal loss, reduced ratio of interneurons to the total number of neurons, and reduction in spontaneous inhibitory postsynaptic currents accompanied by increased anxiety were found in the MDZ-treated group. The rats treated with LY293558 did not differ from the control rats (not exposed to soman) in any of these measurements. Thus, LY293558 has significantly greater efficacy than midazolam in protecting against prolonged seizures and brain damage caused by acute nerve agent exposure.

## 1. Introduction

Acute nerve agent exposure affects both the peripheral and the central nervous system, and can result in an agonizing death if not treated in a timely manner. The primary action of these organophosphorus agents is the inhibition of acetylcholinesterase; the resulting elevation of acetylcholine and the hyperstimulation of cholinergic receptors are the main causes of both the peripheral and central effects [1]. Rapid death can ensue, due to respiratory failure caused by bronchospasms and thick secretions in the airways, weakness and eventual paralysis of the respiratory muscles, and suppression of the respiratory center [2]. These effects can be prevented or adequately controlled by the prompt administration (or auto-injection) of available and efficacious drugs, such as atropine, which counteracts the excessive activation of muscarinic cholinergic receptors, primarily in the peripheral nervous system. In the brain, acute nerve agent exposure induces unrelenting status epilepticus (SE), which can also lead to death. If death is prevented but SE is not adequately controlled, the brain will be damaged by the intense seizures, which will result in long-term neurological and behavioral deficits [3]. Therefore, control of the seizures after acute nerve agent exposure is necessary to prevent death or brain damage.

The seizures induced by nerve agent exposure are initiated primarily due to hyperstimulation of muscarinic receptors by the elevated acetylcholine [4,5,6,7,8]. The hyperstimulation of the muscarinic receptors triggers excessive activity in the glutamatergic synapses [9,10,11], which reinforces and sustains seizures [12], and is the main cause of excitotoxic neuronal damage and death [13,14]. Excessive glutamatergic excitation can be suppressed by enhancing the GABA_A_ receptor-mediated inhibitory activity. Benzodiazepines—mainly diazepam (DZP) and midazolam (MDZ) which are positive modulators of GABA_A_ receptors [15,16,17]—are most often used for the suppression of seizures and are administered as the first-line treatment for SE, regardless of the etiology [18,19,20]. However, seizures often reoccur after a transient suppression by the administration of DZP or MDZ [21,22,23]. Furthermore, benzodiazepines may be ineffective if administered at delayed time points after the onset of SE, and for this reason, they are recommended for early treatment [18,20,24]. There are also cases where the seizures are completely refractory to benzodiazepines [18,20,25].

Some of these drawbacks have also been observed when benzodiazepines are administered to terminate SE induced by nerve agents, in animal models of nerve agent exposure. Thus, the anticonvulsant efficacy of DZP decreases as the latency from the onset of SE induced by the nerve agent soman increases [26,27,28]. In addition, the seizures induced by exposure to soman or other organophosphorus agents return after a temporary cessation by administration of DZP [29,30]. More importantly, cessation of nerve agent-induced SE by DZP does not prevent brain damage, or the appearance of behavioral and neurological deficits [29,31,32,33].

Despite this knowledge, DZP is the current FDA-approved anticonvulsant for treating the victims of acute nerve agent exposure. MDZ has a more rapid absorption and onset of action than DZP, as well as greater water solubility and a longer shelf life [34]; for these reasons, the FDA is currently considering the approval of MDZ for the treatment of nerve agent-induced SE. However, it is unclear if, in the same way as DZP, MDZ is not a good neuroprotectant, particularly if administered with some delay after the exposure. The present study was undertaken to enhance knowledge of the antiseizure and neuroprotective efficacy of MDZ when used to control nerve agent-induced SE. The efficacy of MDZ was compared with that of a compound that directly counteracts glutamatergic hyperexcitation, the AMPA/GluK1 receptor antagonist (3*S*, 4*aR*, 6*R*, 8*aR*)-6-[2-(1(2)*H*-tetrazole-5-yl)ethyl]decahydroisoquinoline-3-carboxylic acid (LY293558 [35]; also known as Tezampanel), which we previously tested against soman under various experimental conditions and obtained promising results [36,37,38,39,40].

## 2. Materials and Methods

### 2.1. Animals

Sprague-Dawley male rats, purchased from Charles River Laboratories (Wilmington, MA, USA), were individually housed in an environmentally controlled room (20–23 °C, 12-h light/12-h dark cycle, lights on 06:00 a.m.), with food and water available ad libitum. The rats weighed 150–250 g at the start of the experiments. The experiments performed followed the Guide for the Care and Use of Laboratory Animals (Institute of Laboratory Animal Resources, National Research Council), and were approved by the Institutional Animal Care and Use Committees of the Uniformed Services University of the Health Sciences and the U.S. Army Medical Research Institute of Chemical Defense (Approval number APG-18-677). The animal care and use programs of both of the institutions are accredited by the Association for Assessment and Accreditation of Laboratory Animal Care International.

### 2.2. Soman Administration and Drug Treatment

Soman (pinacolyl methylphosphonofluoridate) was obtained from Edgewood Chemical Biological Center (Aberdeen Proving Ground, MD, USA); it was diluted in cold saline and administered via a single subcutaneous injection (132 µg/kg, 1.2 × LD_50_). To alleviate the peripheral effects of soman we injected the rats with 2 mg/kg atropine sulfate (IM; Sigma-Aldrich, St. Louis, MO, USA), a muscarinic receptor antagonist, and 125 mg/kg HI-6 (IP; Starks Associates, Buffalo, NY, USA), a bispyridinium oxime that reactivates inhibited AChE, primarily in the periphery [41], within 1 min after injection of soman. One hour after the soman exposure, the rats were injected (IM) with either MDZ (5 mg/kg; Hospira Inc., Lake Forest, IL, USA) or LY293558 (10 mg/kg; kindly provided by Raptor Pharmaceutical Corp., Novato, CA, USA). In deciding the appropriate anticonvulsant dose to use, we aimed for the lowest dose that will completely stop seizure activity in less than 30 min (Figure S1), as high anticonvulsant doses may contribute to cardiorespiratory depression, particularly in an animal undergoing SE. In the present study, we did not use a group of rats that was exposed to soman but did not receive any anticonvulsant treatment because (1) we have obtained such data in previous studies [29,36,37,38,42,43,44,45] and, therefore, considered it unnecessary to have more animals experiencing prolonged, unalleviated SE; and (2) the focus of the present study was to compare the outcomes when soman-exposed rats are treated with LY293558 versus MDZ.

The rats, implanted with electrodes for electroencephalographic (EEG) recordings, were exposed to soman two weeks after the electrode implantation. After the exposure, all of the rats (electrode implanted and non-implanted) were injected with 5 mL Lactate Ringer’s solution every 8 h for 24 h, which continued for another 24 h in the animals that had not completely recovered and appeared to need more hydration. The rats implanted with EEG electrodes were euthanized after the completion of 24 h of recording. For the evaluation of neuropathology and anxiety-like behavior, only the rats that were not implanted with electrodes were used, so as to avoid potential confounded variables (for example, additional stress in the electrode-implanted rats, arising from the presence of the headpiece and the electrodes, particularly during SE).

### 2.3. Electrode Implantation and EEG Recordings

**Figure 1:**
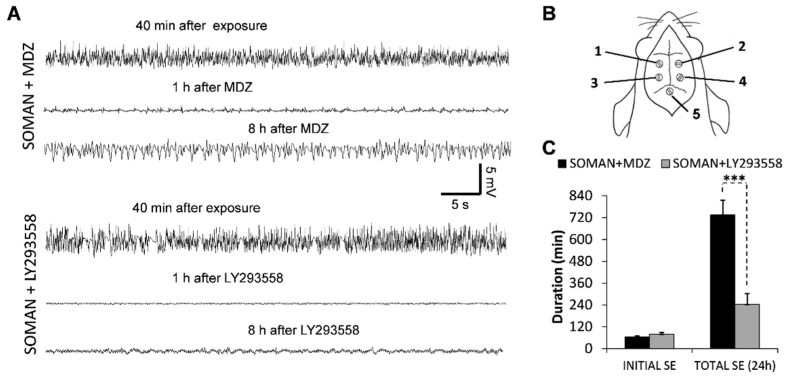
Intramuscular administration of midazolam (MDZ) or LY293558 stopped soman-induced SE within less than 30 min of administration, but seizures reoccurred significantly more in the MDZ-treated group. MDZ (5 mg/kg) or LY293558 (10 mg/kg) were administered at 1 h after injection of soman (1.2 × LD_50_). Representative EEG traces at 40 min after soman exposure, as well as at 1 h and 8 h after administration of MDZ or LY293558 are shown in (**A**). Notice the persistence of low-amplitude epileptiform activity and the return of seizures in the MDZ-treated rats, but not in the LY293558-treated rats. Electrode placement for the recordings is shown diagrammatically in (**B)** (one, two, three, and four, parietal electrodes; five, cerebellar reference electrode). The group data in (**C**) show the duration of the initial SE in each of the two groups and the total duration of SE throughout the 24 h period after soman exposure (*** *p* < 0.001, Independent Samples *t*-test). Sample sizes are *n* = eight for each of the two groups.

The rats were anesthetized with isoflurane using a gas anesthesia system (Kent Scientific, Torrington, CT, USA), and five stainless steel, cortical screw electrodes were stereotaxically implanted, as previously described (Figure 1B) [29]. The video-EEG recordings were obtained in the freely-moving rats at a sampling rate of 200 Hz, using an EEG system (Stellate, Montreal, QC, Canada). The recordings were visually analyzed offline with the filters set to 0.3 Hz for the low frequency filter, 60 Hz for the notch filter, and 70 Hz for the high frequency filter, using the Harmonie Viewer 6.1e from Stellate (Montreal). The EEG recordings were obtained starting 1 to 2 h before the soman exposure and continuing for another 24 h after the soman injection. The disappearance of large amplitude, repetitive discharges (>1 Hz with at least double the amplitude of the background activity) was considered to be evidence of cessation of SE.

### 2.4. Preparation of Brain Sections

The neuropathological analysis was performed in the basolateral amygdala (BLA), 30 days after the exposure. The rats were deeply anesthetized with pentobarbital (75–100 mg/kg, i.p.) and transcardially perfused with PBS (100 mL), followed by 4% paraformaldehyde (200 mL). The brains were removed and placed in 4% paraformaldehyde, overnight at 4 °C, for post-fixation. The next day, the brains were transferred to a solution of 30% sucrose in PBS for 72 h, and frozen with dry ice before storage at −20 °C, until sectioning. A one-in-five series of sections (for every five sections cut in series, one was kept), starting from the rostral extent of the amygdala, were cut at 40 µm on a sliding microtome and mounted on Superfrost Plus slides (Daigger, Vernon Hills, IL, USA). Two adjacent sections from every five were used, one for Nissl staining with Cresyl Violet and one for immunohistochemical labeling for GAD-67. The neuropathological evaluation was completed in a blind fashion.

### 2.5. GAD-67 Immunohistochemistry

Our GAD-67 immuno-labeling procedure was previously described [36,44]. The sections were collected from the cryoprotectant solution, washed three times for 5 min each in 0.1 M PBS, and then kept for 1 h in a solution containing 10% normal goat serum (Chemicon, CA, USA) and 0.5% Triton X-100 in PBS, at room temperature. The sections were then incubated with mouse anti-GAD-67 serum (1:1000, MAB5406; Chemicon), 5% NGS, 0.3% Triton X-100, and 1% bovine serum albumin, overnight at 4 °C. After rinsing three times for 10 min each in 0.1% Triton X-100 in PBS, the sections were incubated with Cy3-conjugated goat anti-mouse antibody (1:1000; Jackson ImmunoResearch, West Grove, PA, USA) and 0.0001% DAPI (Sigma, St. Louis, MO, USA) in PBS, for 1 h at room temperature. Subsequently, the sections were rinsed in PBS for 10 min, mounted on slides, air dried for 30 min, and cover-slipped with ProLong Gold antifade reagent (Invitrogen, Waltham, MA, USA).

### 2.6. Estimation of Neuronal and Interneuronal Loss

Design-based stereology was used to quantify the total number of neurons in the Nissl-stained sections, and the interneurons in the GAD-67 immuno-stained sections, in the BLA, as previously described [36,44]. The sections were viewed with a Zeiss Axioplan 2ie fluorescent microscope (Oberkochen, Germany), equipped with a motorized stage, and interfaced with a computer running StereoInvestigator 8.0 (MicroBrightField, Williston, VT, USA). The BLA was identified under a 2.5× objective on slide-mounted sections, using as the reference the atlas of Paxinos and Watson, 2005 [46]. All of the counting was completed under a 63× oil immersion objective. The total number of Nissl-stained and GAD-67-immunostained neurons was estimated, using the optical fractionator probe, and, along with the coefficient of error (CE), were calculated using the Stereo Investigator 8.0 (MicroBrightField, Williston, VT, USA). The CE was calculated by the software, according to the Gundersen (m = 1; [47]) and Schmitz–Hof (second estimation; [48]) equations.

To determine the number of Nissl-stained neurons in the BLA, one section in a series of five sections was analyzed (seven sections were used on average from each rat). The counting frame was 35 × 35 µm, the counting grid was 190 × 190 µm, and the dissector height was 12 µm. The nuclei were counted when the cell body came into focus within the dissector, which was placed 2 µm below the section surface. The section thickness was measured at every counting site, and the average mounted section thickness was 20 µm. An average of 345 neurons per rat was counted, and the average CE was 0.05 for both the Gundersen and Schmitz–Hof equations.

To determine the number of neurons immuno-labeled for GAD-67, one section in a series of 10 sections was analyzed (on average, five sections from each rat). The counting frame was 60 × 60 µm, the counting grid was 100 × 100 µm, and the dissector height was 20 µm. The nuclei were counted when the top of the nucleus came into focus within the dissector, which was placed 2 µm below the section surface. The section thickness was measured at every fifth counting site, and the average mounted section thickness was 27 µm. An average of 235 neurons per rat was counted, and the average CE was 0.08 for both the Gundersen and Schmitz–Hof equations.

### 2.7. Electrophysiological Experiments

The procedures followed to obtain the whole-cell recordings from the BLA have been previously described [49]. The rats were anesthetized with isoflurane before decapitation. The coronal brain slices (400 µm-thick) containing the amygdala were cut in ice-cold solution (consisting in mM: 115 sucrose; 70 NMDG; 1 KCl; 2 CaCl_2_; 4 MgCl_2_; 1.25 NaH_2_PO_4_; 30 NaHCO_3_; 25 d-glucose) with the use of a vibratome (Leica VT 1200 S; Leica Microsystems, Buffalo Grove, IL, USA). The slices were transferred to a holding chamber at room temperature, in a bath solution containing (in mM): 125 NaCl; 2.5 KCl; 1.25 NaH_2_PO_4_; 21 NaHCO_3_; 2 CaCl_2_; 1 MgCl_2_; and 11 D-glucose. The recording solution (artificial cerebrospinal fluid; ACSF) was the same as the holding bath solution. All of the solutions were saturated with 95% O_2_/5% CO_2_ to achieve a pH near 7.4. The recording chamber (0.7 mL capacity) had continuously flowing ACSF (~8 mL/min) at 30 to 31 °C. The osmolarity of the ACSF was adjusted to 325 mOsm with D-glucose.

To visualize the neurons in the BLA, we used a 40× water immersion objective equipped with a CCD-100 camera (Dage-MTI, Michigan City, IN, USA), under infrared light, using Nomarski optics of an upright microscope (Zeiss Axioskop 2, Thornwood, NY, USA). The recording electrodes had resistances of 3.5~4.5 MΩ when filled with the internal solution (in mM): 60 CsCH_3_SO_3_; 60 KCH_3_SO_3_; 5 KCl; 10 EGTA; 10 HEPES; 5 Mg-ATP; 0.3 Na_3_GTP (pH 7.2; osmolarity was adjusted to 290 mOsm with potassium gluconate). Tight-seal (over 1 GΩ) whole-cell recordings were obtained from the cell body of the principal neurons, distinguished from the interneurons by their larger size, pyramidal shape, and electrophysiological characteristics [49,50,51,52]. Access resistance (5~24 MΩ) was regularly monitored during the recordings, and the cells were rejected if the resistance changed by more than 15% during the experiment.

The currents were amplified and filtered (1 kHz) using the Axopatch 200B amplifier (Axon Instruments, Foster City, CA, USA) with a four-pole, low-pass Bessel filter, digitally sampled (up to 2 kHz) using the pClamp 10.5 software (Molecular Devices, Sunnyvale, CA, USA), and subsequently analyzed using the Mini Analysis program (Synaptosoft Inc., Fort Lee, NJ, USA) and Origin (OriginLab Corporation, Northampton, MA, USA).

### 2.8. Behavioral Experiments

Anxiety-like behavior in the open field and the responses to acoustic startle were examined 30 days after soman exposure. The behavioral tests used were previously described [44,51]. The open field apparatus consisted of a clear Plexiglas arena (40 × 40 × 30 cm). One day prior to testing (on day 29 after soman exposure), the animals were acclimated to the apparatus for 20 min. On the test day, the rats were placed in the center of the open field, and activity was measured and recorded for 20 min, using an Accuscan Electronics infrared photocell system (Accuscan Instruments Inc., Columbus, OH, USA). The automatic collection of the data was completed with a computer equipped with Fusion software (Accuscan). Locomotion (distance traveled in cm), total movement time, and time spent in the center of the open field were analyzed. Anxiety-like behavior was measured as the ratio of the time spent in the center over the total movement time, expressed as a percentage of the total movement time.

The responses to acoustic startle were assessed, using the Med Associates Acoustic Response Test System (Med Associates, Georgia, VT, USA). This system consists of weight-sensitive platforms inside individual sound-attenuating chambers, and includes a ventilating fan to provide background noise. Each rat was individually placed in a ventilated holding cage. The holding cages were small enough to restrict extensive locomotion, but large enough to allow the subject to turn around and make other small movements. Each cage was placed on a weight-sensitive platform. Movements in response to acoustic stimuli were measured as a voltage change by a strain gauge inside each platform. The rats were placed in the apparatus for two sessions, on post-soman days 28 and 29, for acclimation. The startle stimuli consisted of 110- or 120 dB noise bursts (burst duration 20 ms). Each stimulus had a 2 ms rise and decay time, such that the onset and offset were abrupt, which is a primary requirement for startle. Each trial type (110 dB or 120 dB stimulus) was presented eight times. The trial types were presented in random order to avoid effects and habituation, and the inter-trial intervals ranged randomly from 15 to 25 s. An interfaced Pentium computer, with the Med Associates software, recorded startle amplitude as the difference between the maximal voltage change during the startle period and the maximal voltage change during the no-stimulus periods, and assigned a value based on an arbitrary scale used by the software of the test system.

### 2.9. Statistical Analysis

A Fisher exact test was used to compare the survival rate between the two soman-exposed groups treated with MDZ or LY293558. The other variables of the study were tested for normal distribution, using the Kolgomorov–Smirnov normality test, and all of the results showed a normal distribution (*p* > 0.05). Thus, the Independent Samples *t*-test was used to determine whether there were significant differences between the two groups (treated with MDZ or LY293558) in the time it took for cessation of seizures after anticonvulsant administration, the duration of the initial SE, and the total duration of SE within 24 h after soman exposure. Before comparing the differences between the three groups (soman-exposed group treated with MDZ, soman-exposed group treated with LY293558, and control group not exposed to soman) using analysis of variance (ANOVA), we tested for homogeneity of the variances between the groups, using Levene’s test. The only variable that showed unequal variances (*p* = 0.0002) was the “Total Charge transferred by sIPSCs” (in the electrophysiological experiments); therefore, we used Welch’s ANOVA followed by Games–Howell post hoc test for comparisons of these results. The ANOVA followed by the Bonferroni post hoc test was used to compare stereological estimations of the total number of neurons and interneurons, and ANOVA followed by Holm–Šídák was used to compare the results from the behavioral tests. The statistical values are presented as mean and standard error of the mean. For all of the tests, differences were considered significant when *p* < 0.05. The sample sizes (*n*) refer to the number of animals, except for the in vitro experiments, where *n* refers to the number of recorded neurons.

## 3. Results

### 3.1. Seizure Termination by MDZ or LY293558 and Survival Rates

The male rats implanted with electrodes for monitoring electrographic seizure activity were exposed to soman; the rats were treated with atropine and HI-6 within 1 min after the soman injection, and were administered 5 mg/kg MDZ (*n* = 10, SOMAN + MDZ group) or 10 mg/kg LY293558 (*n* = 11, SOMAN + LY293558 group), 1 h later. The status epilepticus (SE) developed within an average of 9 min after the injection of soman (8.75 ± 0.79 and 9.25 ± 0.75 min for the SOMAN + MDZ and the SOMAN + LY293558 group, respectively). The seizures were completely suppressed within 19.75 ± 1.8 min after administration of the MDZ, and within 29.5 ± 1.75 min after administration of the LY293558 (significantly longer in the LY293558 group, *p* = 0.0017). Thus, the duration of the initial SE (the SE triggered by soman injection and ceased by anticonvulsant administration) was 65 ± 5 min in the SOMAN + MDZ group and 80 ± 9 min in the SOMAN + LY293558 group (*p* = 0.0729, *n* = eight in each group; two rats in the MDZ group and three rats in the LY293558 group died during SE). Monitoring the seizure activity for 24 h after the soman exposure revealed that SE returned after administration of either MDZ or LY293558, but to a significantly greater extent in the MDZ-treated group (representative samples of EEG activity at different time points after soman exposure are shown in Figure 1A). Thus, the total duration of SE within the 24 h post-exposure period was 735 ± 80 min in the SOMAN + MDZ group and 244 ± 59 min in the SOMAN + LY293558 group (*p* = 0.0002; Figure 1C).

Among the animals implanted with electrodes, the 24 h survival rate was 80% (8/10) in the SOMAN + MDZ group and 72.7% (8/11) in the SOMAN + LY293558 group (Fisher exact probability test *p* = 0.77). However, the survival rate among the animals not implanted with electrodes was 100% in both the SOMAN + MDZ and the SOMAN + LY293558 group. The results are summarized in Table 1.

### 3.2. LY293558 but Not MDZ Provides Full Protection against Neuronal and Interneuronal Loss in the BLA

Only the rats that were not implanted with EEG electrodes were used for neuropathology and behavioral testing. Thirty days after soman exposure, the total number of neurons in the BLA was estimated, using an unbiased stereological method in the Nissl-stained sections. The BLA was selected to study both the pathology and pathophysiology because this amygdala nucleus plays a central role in seizure initiation and propagation after nerve agent exposure [53,54,55], and the amygdala is one of the brain regions where nerve agents cause the most severe neuropathology [42].

**Figure 2:**
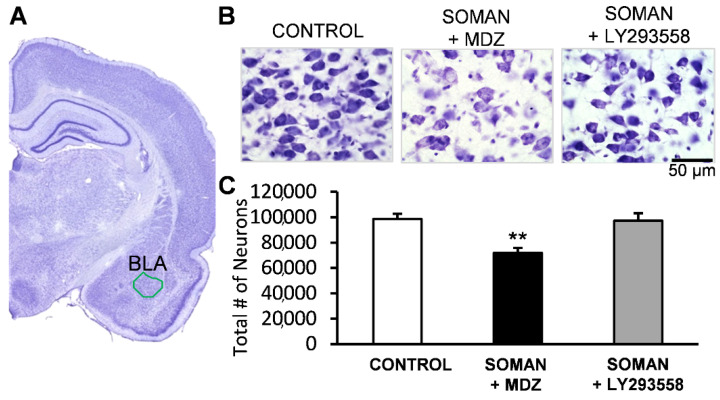
Thirty days after soman exposure, there was significant neuronal loss in the BLA of the MDZ-treated group but not the LY293558-treated group. (**A**) Panoramic view of a Nissl-stained section showing the area where neuronal loss was assessed; (**Β**) Representative photomicrographs from a control (not exposed) rat and an MDZ- or LY293558-treated soman-exposed rat (total magnification 630×; scale bar, 50 μm); (**C**) Group data; sample sizes: *n* = 8 for each group; ** *p* < 0.01 compared with either the control or the SOMAN + LY293558 group (ANOVA, Bonferroni post hoc test).

The number of neurons in the BLA of the SOMAN + MDZ group (71,771 ± 3980; *n* = 8) was significantly lower than the number of neurons in the BLA of the SOMAN + LY293558 group (97,305 ± 5872; *n* = 8; *p* = 0.003) and a control group that was not exposed to soman (98,587 ± 4287; *n* = 8; *p* = 0.002; Figure 2). The number of neurons in the SOMAN + LY293558 group did not differ from the control (*p* = 0.9).

**Figure 3:**
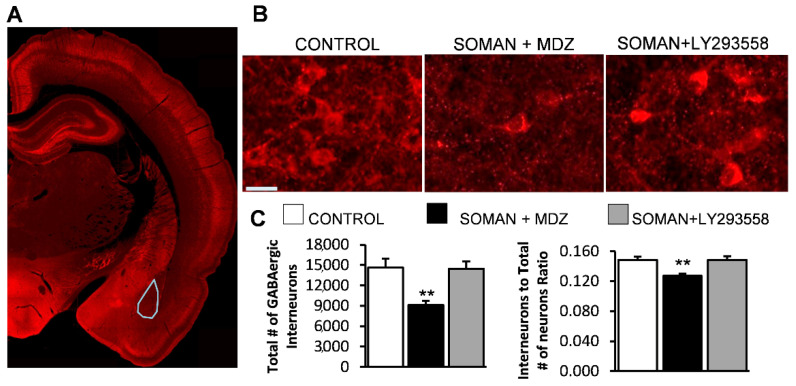
Thirty days after soman exposure, there was significant loss of GABAergic interneurons in the BLA of the MDZ-treated group but not the LY293558-treated group. (**A**) Panoramic view of a GAD-67 immuno-stained section showing the area where interneuronal loss was assessed; (**B**) Representative photomicrographs of GAD-67 immuno-stained interneurons in the BLA from a control (not exposed) rat and an MDZ- or LY293558-treated soman-exposed rat (total magnification 630×; scale bar, 50 μm); (**C**) Group data of the total number of GABAergic interneurons (left panel) and the ratio of the number of GABAergic interneurons to the total number of neurons (right) for the two experimental groups and a control group. Sample sizes: *n* = 8 for each group; ** *p* < 0.01 compared with either the control or the SOMAN + LY293558 group (ANOVA, Bonferroni post hoc test).

The estimation of the total number of GABAergic interneurons in the BLA, using an unbiased stereological method on the GAD-67 immuno-stained sections, showed that thirty days after soman exposure the number of interneurons in the BLA of the SOMAN + MDZ group (9125 ± 597, *n* = 8) was significantly lower than that in the SOMAN + LY293558 group (14,466 ± 1070, *n* = 8; *p* < 0.01) and a control group that was not exposed to soman (14,657 ± 1.281, *n* = 8; *p* < 0.01; Figure 3). The ratio of the GABAergic interneurons to the total number of neurons was also significantly lower in the SOMAN + MDZ group (12.7% ± 0.3), compared to either the SOMAN + LY293558 group (14.86% ± 0.47, *p* < 0.01), or the control group (14.86% ± 0.45, *p* < 0.01; Figure 3). The number of interneurons and the ratio of interneurons to the total number of neurons in the BLA of the SOMAN + LY293558 group did not differ from the control (*p* = 0.9).

### 3.3. LY293558 but Not MDZ Prevents a Reduction of Background Inhibition in the BLA

We have previously shown that, along with the loss of interneurons in the BLA after soman-induced SE that is not treated with an anticonvulsant [36,44], there is a reduction in the background inhibitory activity [45]. Therefore, we investigated whether the treatment of soman-exposed rats with MDZ or LY293558 prevented a reduction in the spontaneous IPSCs in the BLA.

**Figure 4:**
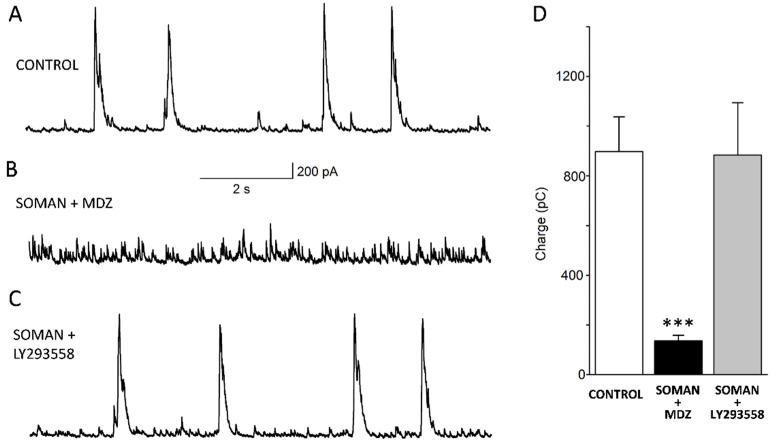
Thirty days after soman exposure, spontaneous inhibitory activity was reduced in the BLA of the MDZ-treated group but not the LY293558-treated group. Representative GABA_A_ receptor-mediated sIPSC current traces recorded from BLA principal neurons from a control rat (**A**) and from soman-exposed rats treated with MDZ (**B**) or LY293558 (**C**). Recordings were obtained at +30 mV holding potential; (**D**) Group data of charge transferred by sIPSCs, during a 10 s time window. *** *p* < 0.001 when inhibitory activity of neurons from the MDZ-treated group (*n* = 14) is compared with the inhibitory activity in either the control (*n* = 14) or the LY293558-treated group (*n* = 8; Welch’s ANOVA followed by Games–Howell post hoc test).

In the BLA of the control rats (not exposed to soman), the principal/pyramidal neurons generated synchronous “bursts” of GABA_A_ receptor-mediated IPSCs (in 12 out of 14 neurons; Figure 4A), which is consistent with previous reports [49,56,57,58]. In the rats exposed to soman and treated with MDZ, this rhythmic synchronous inhibitory activity was absent at 30 days after exposure, in all of the recorded neurons (*n* = 14; Figure 4B). In contrast, the soman-exposed rats treated with LY293558, displayed bursts of sIPSCs (Figure 4C) in six out of eight neurons. To quantify the differences between the groups we calculated the total charge transferred (the area delimited by the inhibitory current and the baseline) in picocoulombs (pC), for a time window of 10 s; we included all of the recorded neurons in these comparisons, whether they generated rhythmic sIPSC bursts or displayed only conventional spontaneous inhibitory activity. The total charge transferred by the sIPSCs in the SOMAN + MDZ group (135.61 ± 22.18 pC; *n* = 14) was significantly different from that in the SOMAN + LY293558 group (883.45 ± 210.29; *n* = 8; *p* < 0.001) and a control group that was not exposed to soman (897.27 ± 139.36 pC; *n* = 14; Figure 4D). The total charge transferred by the sIPSCs in the SOMAN + LY293558 group did not differ from the control (*p* = 0.12).

### 3.4. LY293558 but Not MDZ Prevents an Increase in Anxiety-like Behavior

**Figure 5:**
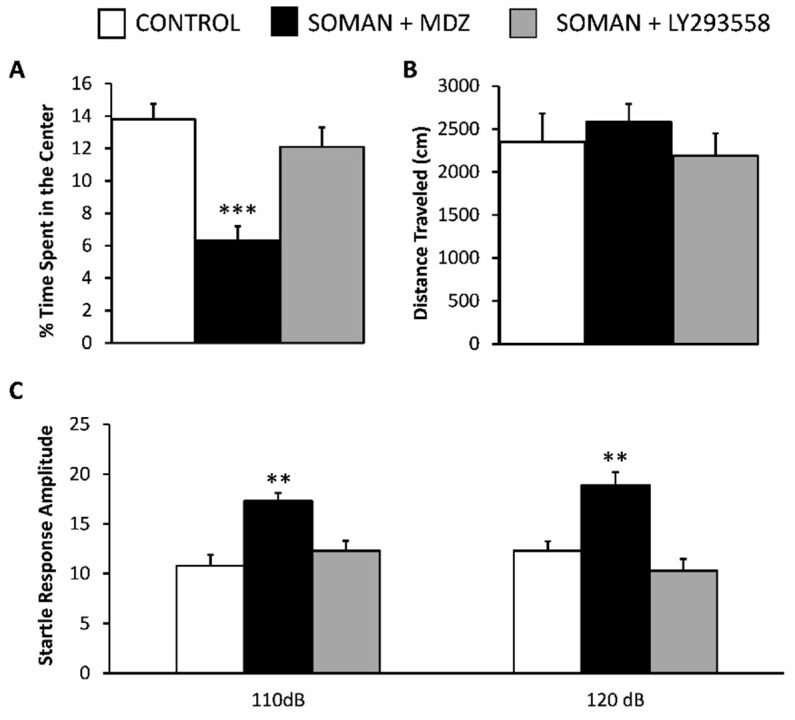
Thirty days after soman exposure, anxiety-like behavior was increased in the MDZ-treated group but not the LY293558-treated group. (**A**) Percentage of time spent in the center of the open field; (**B**) Distance traveled in the open field; (**C**) Amplitude of startle responses to 110- and 120-db acoustic stimuli. Sample sizes: *n* = 8 for each group. Control group is rats not exposed to soman. ** *p* < 0.01, *** *p* < 0.01 (One-Way ANOVA with Holm-Šídák post hoc test).

To investigate whether the neuropathology observed in the BLA of the MDZ-treated rats and the reduction in spontaneous inhibitory activity had translated into behavioral deficits, we examined anxiety-like behavior with the use of two tests, the open field [59] and the acoustic startle response (ASR; [60]). Thirty days after the soman exposure, the SOMAN + MDZ group spent significantly less time in the center of the open field (6.3 ± 0.9% of the total movement time, *n* = 8), when compared with either the SOMAN + LY293558 group (12.1 ± 1.2 % of the total movement time, *n* = 8) or a control group which was not exposed to soman (13.8 ± 0.95 % of the total movement time, *n* = 8; *p* < 0.001 for both comparisons; Figure 5A). The time spent in the center for the SOMAN + LY293558 group did not differ from the control (*p* = 0.5). The distance traveled in the open field did not differ between the control rats (2530 ± 330 cm) and the SOMAN + MDZ (2580 ± 290 cm) or the SOMAN + LY293558 (2189 ± 300 cm) treated groups (*p* = 0.6; Figure 5B).

In the acoustic startle response test, the startle amplitude in response to the 110 dB acoustic stimulus was significantly greater in the SOMAN + MDZ group (17.3 ± 0.8, *n* = 8) compared with either the SOMAN + LY293558 group (12.3 ± 1, *n* = 8) or the control group that was not exposed to soman (10.8 ± 1.1, *n* = 8; *p* < 0.01 for both comparisons; Figure 5C, left set of bars). Similarly, the startle amplitude in response to the 120 dB acoustic stimulus was significantly greater in the SOMAN + MDZ group (18.9 ± 1.3, *n* = 8) than in the SOMAN + LY293558 group (10.3 ± 1.2, *n* = 8) or the control group that was not exposed to soman (12.3 ± 0.95, *n* = 8; *p* < 0.01 for both comparisons; Figure 5C, right set of bars). The startle amplitude in the SOMAN + LY293558 group in response to either 110 or 120 dB did not differ from the control (*p* = 0.53 and *p* = 0.45 for the 110 and 120 dB, respectively).

## 4. Discussion

In this study, we compared the efficacy of MDZ—a benzodiazepine commonly used in the treatment of SE and currently considered by the FDA for approval as a better alternative to DZP for the control of nerve agent-induced SE—with the efficacy of an AMPA/GluK1 receptor antagonist, LY293558 [35], in suppressing seizures and protecting the brain in adult male rats exposed to soman. We found that both MDZ and LY293558 promptly stopped the initial SE, with MDZ suppressing seizures significantly faster than LY293558. In addition, both of the anticonvulsant treatments produced a 100% survival rate. However, the seizures reoccurred significantly more in the MDZ-treated rats; as a result, the total duration of SE in the 24 h period after soman exposure was significantly longer in the MDZ-treated group compared with the LY293558-treated group. Furthermore, when the brain damage was examined 30 days later, it was found that in the BLA of the MDZ-treated group there was a significant loss of interneurons and a reduced total number of neurons. The neuronal and interneuronal loss in the BLA of the MDZ-treated rats was accompanied by reduced GABAergic inhibition and loss of rhythmic GABAergic activity, as well as increased anxiety-like behavior. Such alterations had not occurred in the group treated with LY293558, in which the number of neurons and interneurons in the BLA, GABAergic inhibition, and anxiety-like behavior did not differ from the control rats that were not exposed to soman.

A significant reoccurrence of the seizures after cessation of SE by administration of a benzodiazepine was previously observed in both animals [6,29,30,61] and humans [23,62]. In the present study, the average total duration of SE in the MDZ-treated group within 24 h after soman exposure was 735 min, which is nearly identical to the average duration of SE in same-age male rats exposed to the same dose of soman as in the present study, but without receiving anticonvulsant treatment (609 min total duration of SE; [43]). A plausible explanation for the return of SE after a prompt but transient cessation by MDZ or DZP may be based on the weakening of the GABAergic inhibition. Intense seizure activity induces the internalization of postsynaptic GABA_A_ receptors [63,64,65,66]; this may be caused by the excess GABA being released during synaptic hyperactivity [65,67], and/or mechanisms related to intense NMDA receptor activation [68,69]. When a benzodiazepine is administered, the activity of the GABA_A_ receptors may be enhanced sufficiently to halt seizures. However, the availability of the GABA_A_ receptors is progressively reduced, making these drugs ineffective in sustaining enhanced synaptic inhibition, even more so as they are cleared from the blood. Therefore, glutamatergic hyperexcitability overrides and seizures reoccur.

A reduction in the total number of neurons and a loss of interneurons in the BLA was found in the MDZ-treated group; this is consistent with a previous study in which the extent of the neuropathology in soman-exposed rats who received DZP treatment 1 h after exposure was virtually the same as in the soman-exposed group that did not receive anticonvulsant treatment [29]. The limited or absent neuroprotective efficacy of MDZ has been previously reported, and the timing of MDZ administration appears to be important in this regard. Thus, MDZ protects against nerve agent- or other organophosphate-induced neuropathology in adult or young-adult rats, if it is administered at the time of exposure [70], or at the onset of seizures [71], or after 5 min of seizure activity [72], but not if it is given at 1 h [70,73], 30 min, or 2 h after SE onset [73]. Therefore, it is commonly presumed that the brain damage that is seen when the anticonvulsant administration is delayed is caused primarily by the SE that occurred before the anticonvulsant administration [70,73]; this seems to be a reasonable assumption, particularly if the SE was severe. However, our results suggest that SE lasting up to 1 h does not necessarily produce long-term brain damage, and that the outcome may significantly depend on whether or not the type of the anticonvulsant used can limit the total duration of SE. Thus, in the present study, both MDZ and LY293558 were administered after about 50 min of ongoing SE, both of the anticonvulsants completely suppressed seizures in less than 30 min, but only LY293558 protected from brain damage (at least at the 30-day time point that we examined). In previous studies in immature rats exposed to soman, in which DZP, LY293558, or LY293558 + caramiphen were administered 1 h after soman exposure, all three treatments promptly stopped the initial SE, but only LY293558 and LY293558 + caramiphen protected against brain damage [31]. Similarly, when DZP or UBP302 was administered 1 h after the exposure of adult rats to soman, UBP302 took significantly longer than DZP to stop the initial SE, and yet only UBP302 provided significant neuroprotection [29]. The main observation that is common among these studies is that the seizures reoccurred to a dramatically increased extent after the benzodiazepine treatment. Taken together, these findings suggest that the most important factor determining the extent of the brain damage is the total duration of the SE. The extent of neuroprotection may also depend on the mechanisms of action of the administered anticonvulsant, which may halt the pathological processes triggered by the initial SE, or allow them to progress unopposed.

The reduction in the GABAergic interneurons and total number of neurons in the BLA of the MDZ-treated rats, and the decreased ratio of the number of interneurons to the total number of neurons, were accompanied by a reduction in the spontaneous inhibitory activity. Remarkably, there was a loss of the spontaneous “bursts” of GABA_A_ receptor-mediated IPSCs that are typically generated by the principal neurons in the BLA, in an oscillatory fashion, at an average frequency of 0.5 Hz [49,56,57,58]. This synchronous, rhythmic inhibitory activity, which was found to be triggered by the rhythmic burst firing of the interneurons dependent on the GluN2A-NMDA receptors [49] and ASIC1a channels [58], may play an important role in the oscillatory activity patterns generated by the BLA network; these have been associated with cognitive and emotional functions, such as the perception of safety [74,75], and the expression of fear [76]. Therefore, the loss of this inhibitory bursting in the BLA of the MDZ-treated rats may have implications for cognitive and/or emotional behavior. Accordingly, in the present study, the MDZ-treated group displayed increased anxiety-like behavior. The level of increase in anxiety in the MDZ-treated rats, as determined with the use of the open field and the acoustic startle response tests (6.3% of total movement time was spent in the center of the open field, while the average startle amplitude was about 18), is almost identical to that obtained from same-age male rats exposed to the same dose of soman as in the present study, but without receiving anticonvulsant treatment (6.7% of total movement time was spent in the center of the open field and the average startle amplitude was about 18; [37]), indicating the minimal neuroprotective effects of this drug.

## 5. Conclusions

In the event of a mass exposure to nerve agents, there will likely be a delay before the victims are under medical care and monitoring. The administration of MDZ with some delay after the onset of SE can be expected to save lives by controlling the initial SE, but it will not protect against brain damage and long-term morbidity. Perhaps a higher dose of MDZ, or repeated injections of MDZ to suppress the reoccurrence of seizures —thus limiting the total duration of SE—could have a better outcome on neuroprotection, but clinical experience clearly points to the need for anticonvulsants other than MDZ or DZP for the long-term management of SE. Furthermore, if the victims of nerve agent exposure are very young, when the GABAergic system in the brain is still immature and GABA_A_ receptor activation may produce depolarization [77,78,79], MDZ administration could be detrimental [80]. The glutamate receptor antagonists, such as LY293558, do not only stop the initial SE and decrease mortality rate, but also provide significant protection against brain damage. Assessment of brain damage at longer post-exposure time points (beyond 30 days after soman exposure) will confirm whether the neuroprotection by LY293558 after acute nerve agent exposure can be considered lifelong.

## Figures and Tables

**Table 1:** Effects of MDZ (5 mg/kg, i.m) or LY293558 (10 mg/kg, i.m) on soman-induced status epilepticus (SE) and survival rate, when the anticonvulsants are administered 1 h after soman exposure.

Experimental Groups	SOMAN + MDZ	SOMAN + LY293558
Time to cessation of the initial SE (min)	19.75 ± 1.8	29.5 ± 1.75 **
Duration of the initial SE (min)	65 ± 5	80 ± 5.9
Total duration of SE in 24 h (min)	735 ± 80	244 ± 59 ***
Survival rate: electrode-implanted rats	80% (8/10)	72.7% (8/11)
Survival rate: non-implanted rats	100% (11/11)	100% (11/11)

** *p* < 0.01, significantly longer compared with the MDZ group; *** *p* < 0.001, significantly shorter compared with the MDZ group (Independent Samples *t*-test).

## Data Availability

The data that support the findings of this study are available from the corresponding author upon reasonable request.

## Supplementary Materials

The following supporting information can be downloaded at: https://www.mdpi.com/article/10.3390/toxics10080409/s1, Figure S1: Dose-response graphs showing the time to cessation of SE after administration of different doses of midazolam (MDZ) or LY293558.

## Abbreviations

BLA, basolateral amygdala; DZP, diazepam; LY293558, (3S,4aR,6R,8aR)-6-[2-(1(2)H-tetrazole-5-yl)ethyl]decahydroisoquinoline-3-carboxylic acid; HI-6, (1-(2-hydroxyiminomethylpyridinium)-3-(4-carbamoylpyridinium)-2-oxapropane dichloride; MDZ, midazolam; SE, status epilepticus.
