# Supplementary material for: Antiseizure and Neuroprotective Efficacy of Midazolam in Comparison with Tezampanel (LY293558) against Soman-Induced Status Epilepticus
Source: Toxics. 2022 Jul 22;10(8):409. doi: 10.3390/toxics10080409 (PMC9330837; doi:10.3390/toxics10080409)
Supplement: Supplementary file 1 [file toxics-10-00409-s001.zip › toxics-1815384-supplementary.pdf]

toxics

MDPI

Supplementary Materials: Antiseizure and Neuroprotective Efficacy of Midazolam in Comparison with Tezampanel (LY293558) against Soman-Induced Status Epilepticus

Taiza H. Figueiredo, Vassiliki Aroniadou-Anderjaska, Volodymyr I. Pidoplichko, James P. Apland and

Maria F. M. Braga

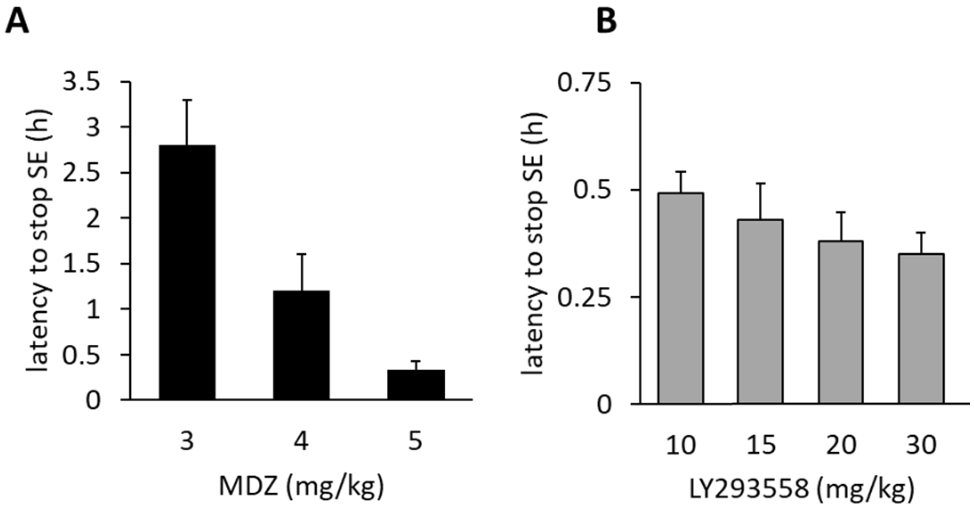

Figure S1. Dose-response graphs showing the time to cessation of SE after administration of different doses of midazolam (MDZ) or LY293558. The anticonvulsants were injected intramuscularly 1 h after exposure of young-adult male rats to soman (single subcutaneous injection of 132 µg/kg, 1.2 X LD50). (A) Bars show the time it took for 3 mg/kg (n = 8), 4 mg/kg (n = 8) and 5 mg/kg (n = 8) of MDZ to stop the initial SE. (B) Bars show the time it took for 10 mg/kg (n = 8), 15 mg/kg (n = 8), 20 mg/kg (n = 9) and 30 mg/kg (n = 10) of LY293558 to stop the initial SE. In the present study, we used 5 mg/kg MDZ and 10 mg/kg LY293558, as both doses stopped SE in less than 30 min and produced 100% survival rate.
